# FixThePig: a custom 3D-printed femoral intramedullary nailing for preclinical research applications

**DOI:** 10.3389/fbioe.2024.1478676

**Published:** 2024-10-17

**Authors:** Julie Manon, Alexandre Englebert, Robin Evrard, Thomas Schubert, Olivier Cornu

**Affiliations:** ^1^ Neuro Musculo Skeletal Lab (NMSK), Institut de Recherche Expérimentale et Clinique (IREC), Université Catholique de Louvain (UCLouvain), Bruxelles, Belgium; ^2^ Service de Chirurgie Orthopédique et Traumatologique, Cliniques Universitaires Saint-Luc, Bruxelles, Belgium; ^3^ Unité de Thérapie Tissulaire et Cellulaire de l’Appareil Locomoteur, Cliniques Universitaires Saint-Luc, Bruxelles, Belgium; ^4^ Institute for Information and Communication Technologies, Electronics and Applied Mathematics (ICTEAM), Electrical Engineering Department (ELEN), UCLouvain, Louvain-la-Neuve, Belgium

**Keywords:** preclinical model, large animal model, critical-size bone defect, custom-made osteosynthesis, intramedullary nailing, femoral nailing, 3D-printing, cutting-guide

## Abstract

**Background:**

Critical-size bone defects (CSBDs) pose significant challenges in clinical orthopaedics and traumatology. Developing reliable preclinical models that accurately simulate human conditions is crucial for translational research. This study addresses the need for a reliable preclinical model by evaluating the design and efficacy of a custom-made 3D-printed intramedullary nail (IMN) specifically for CSBDs in minipigs. The study aims to answer the following questions: Can a custom-made 3D-printed IMN be designed for femoral osteosynthesis in minipigs? Does the use of the custom-made IMN result in consistent and reproducible surgical procedure, particularly in the creation and fixation of CSBDs? Can the custom-made IMN effectively treat and promote bone consolidation of CSBDs?

**Hypothesis:**

The custom-made 3D-printed IMN can be designed to effectively create, fix and treat CSBDs in minipigs, resulting in consistent surgical outcomes.

**Materials and Methods:**

The IMN was designed based on CT scans of minipig femurs, considering factors such as femoral curvature, length, and medullary canal diameters. It was 3D-printed in titanium and evaluated through both *in vitro* and *in vivo* testing. Female Aachen minipigs underwent bilateral femoral surgeries to create and fix CSBDs using the custom-made IMN. Post-operative follow-up included X-rays and CT scans every 2 weeks, with manual examination of explanted femurs to assess consolidation and mechanical stability after 3 months.

**Results:**

The custom-made IMN effectively fitted the minipig femoral anatomy and facilitated reproducible surgical outcomes. Symmetric double osteotomies were successfully performed, and allografts showed minimal morphological discrepancies. However, proximal fixation faced challenges, leading to non-union in several cases, while most distal osteotomy sites achieved stable consolidation.

**Discussion:**

The custom-made 3D-printed IMN demonstrated potential in modelling and treating CSBDs in minipigs. While the design effectively supported distal bone healing, issues with proximal fixation highlight the need for further refinements. Potential improvements include better screw placement, additional mechanical support, and adaptations such as a reduction clamp or a cephalic screw to enhance stability and distribute forces more effectively.

## 1 Introduction

Critical-size bone defects (CSBDs), defined as osseous gaps exceeding the spontaneous healing capacities within the lifespan of an organism (Dalisson et al., 2021), present significant challenges in clinical orthopaedics and traumatology. These defects often result from severe trauma, infection, tumor resection, or congenital abnormalities, and they necessitate innovative treatment strategies to restore structural integrity and function. In clinical practice, the management of these defects commonly involves many surgical treatments (e.g., induced membrane technique (Masquelet and Begue, 2010; Alford et al., 2021; Masquelet, 2020), bone transport (Dalisson et al., 2021; Nauth et al., 2018; Napora et al., 2018; Zekry et al., 2019), bone grafts (Giannoudis et al., 2005; Leemrijse et al., 2006; Delloye et al., 2007)). However, all these interventions often face limitations (Vincken et al., 2023). Research to improve these treatments and reduce their complications is of prime interest. However, the translation between *in vitro* and *in vivo* studies on small animals, which are the most widespread, toward humans is not as obvious. Accurate preclinical models are indispensable for translational research. It is therefore necessary to develop models, particularly those involving large animals, as close as possible to the clinical conditions. Large animal models offer several advantages, including anatomical and biomechanical similarities to human bones (Aerssens et al., 1998; Pearce et al., 2007; Reichert et al., 2009), which are crucial for evaluating the clinical relevance and performance of orthopaedic interventions.

As the Diamond concept highlights, mechanical stability is a key criterion of bone healing (Andrzejowski and Giannoudis, 2019; Giannoudis et al., 2007; Giannoudis et al., 2008). Most preclinical models usually involved plates or external fixators as osteosynthesis. Osteosynthesis by plate and screws was the one previously experimented to fix similar porcine CSBDs (Schubert et al., 2013). The stability was achieved by two orthogonal plates on the femoral shaft. All the studies on osteogenic membranes such as the Masquelet’s technique (Masquelet and Begue, 2010; Masquelet et al., 2000; Klein et al., 2020) or artificial/biological off-the-shelf membranes (Hsu et al., 2020; Manon et al., 2022; Lu et al., 2023; Manon et al., 2023) may be biased when the plate fixation compresses the (a-) cellular membrane and could benefit from another fixation to keep free the additional surrounded scaffold (Klein et al., 2020). Though an easy-to-use device offering a real fixation opportunity for a tibia, external fixator application is not suitable for large animal femurs, which are localized at belly height with an imposing soft-tissue mass. The risks of insufficient stability away from the CSBD, Schantz screw loosening, or pin track infections (Reichert et al., 2009; Klein et al., 2020) are too substantial to consider this option in some cases. The intramedullary nail (IMN), a metallic rod inside the medullary cavity of a long bone, is now very widely used in clinical practice to treat diaphyseal bone fracture. IMN has been a cornerstone in the treatment of human long bone fractures due to its minimally invasive nature, mechanical stability, central biomechanical transmission, load-bearing capacity, and ability to promote early mobilization and weightbearing (Kuntscher, 1940; Soeur, 1946; Watson-Jones et al., 1950; Bong et al., 2006; Bong et al., 2007; Ricci et al., 2009). Potential complications can be managed through various patient- or surgery-dependent factors (Watson-Jones et al., 1950; Manon et al., 2019; Manon et al., 2020). As with every surgical procedure, physicians have to thoroughly discuss the risks, benefits, and alternatives of IMN during the informed consent process to ensure patients have realistic expectations and to enhance overall care quality (Bolcato et al., 2024). Despite its well-established advantages, the use of IMN in managing CSBDs in large animal models remains underexplored. Despite its recognized potential, the implementation of IMN in the context of CSBDs in large animal models remains underexplored.

In recent years, there has been a notable advancement in the field of additive manufacturing for orthopaedic applications. Specifically, the use of 3D-printing in the production of metallic implants has expanded significantly. This includes applications ranging from pre-contoured plates (Maini et al., 2018), to direct 3D-printing of implants such as osteosynthesis plates (Shuang et al., 2016), arthroplasty (Arabnejad et al., 2017), nails (Honda et al., 2024), truss cages (Tetsworth et al., 2017), or a variety of other implants (Popov et al., 2018). 3D-printed Nickel-Titanium implants have shown sufficient biocompatibility in pigs to be used in bone implants (Naujokat et al., 2022).

This study aims to address the need for a reliable preclinical model by proposing the development of a custom-made 3D-printed IMN specifically designed for CSBDs in large animal models, specifically minipigs. The study aims to answer the following questions:

• Can a custom-made 3D-printed IMN be designed for femoral osteosynthesis in minipigs?

• Does the use of the custom-made IMN result in consistent and reproducible surgical procedures, particularly in the creation and fixation of CSBDs?

• Can the custom-made IMN effectively treat and promote bone consolidation of CSBDs?

## 2 Materials and methods

This tailored IMN was designed based on CT scans of minipig femurs, involving several steps from CT scan analyses and template printing to rigorous *ex vivo* testing, culminating in subsequent *in vivo* experiments.

### 2.1 Femoral anatomy

CT scans allowed the study of the anatomy of porcine femurs. Both femurs of Aachen minipigs ( N=14 , from 7 minipigs) were explanted after another study and scanned using a clinical computed tomography (CT scan, Somatom Definition AS 128 scanner, Siemens Healthineers, Erlangen, Germany). These animals were covered by the local ethics committee (Ref 2020/UCL/MD/027 and A1/UCL/2021-A1). Different measures were reviewed around the proximal and distal metaphyses (medullary dimensions, minimal and maximal cortical thicknesses), as well as the diaphysis (outer and inner diameters, smallest intramedullary diameter, minimal and maximal cortical thicknesses). The femoral bending was also assessed in two orthogonal planes (anteroposterior and mediolateral planes), as well as the femoral length and the distance between the IMN entry point at the greater trochanter and the end of the proximal physis. All the measurements were obtained from scanographic images using the same methodology applied to subsequent measurements, as illustrated in Sections 3.1.1, 3.2.

### 2.2 Device featuring

The custom-made IMN was engineered with respect to multiple aspects including:

• The physiological curvature of the femur;

• The length of the diaphysis;

• The diameter of the medullary canal;

• The screw type;

• The screw number;

• The screw divergence;

• The precise positioning of screws along the length of the nail.

### 2.3 3D design

**FIGURE 1:**
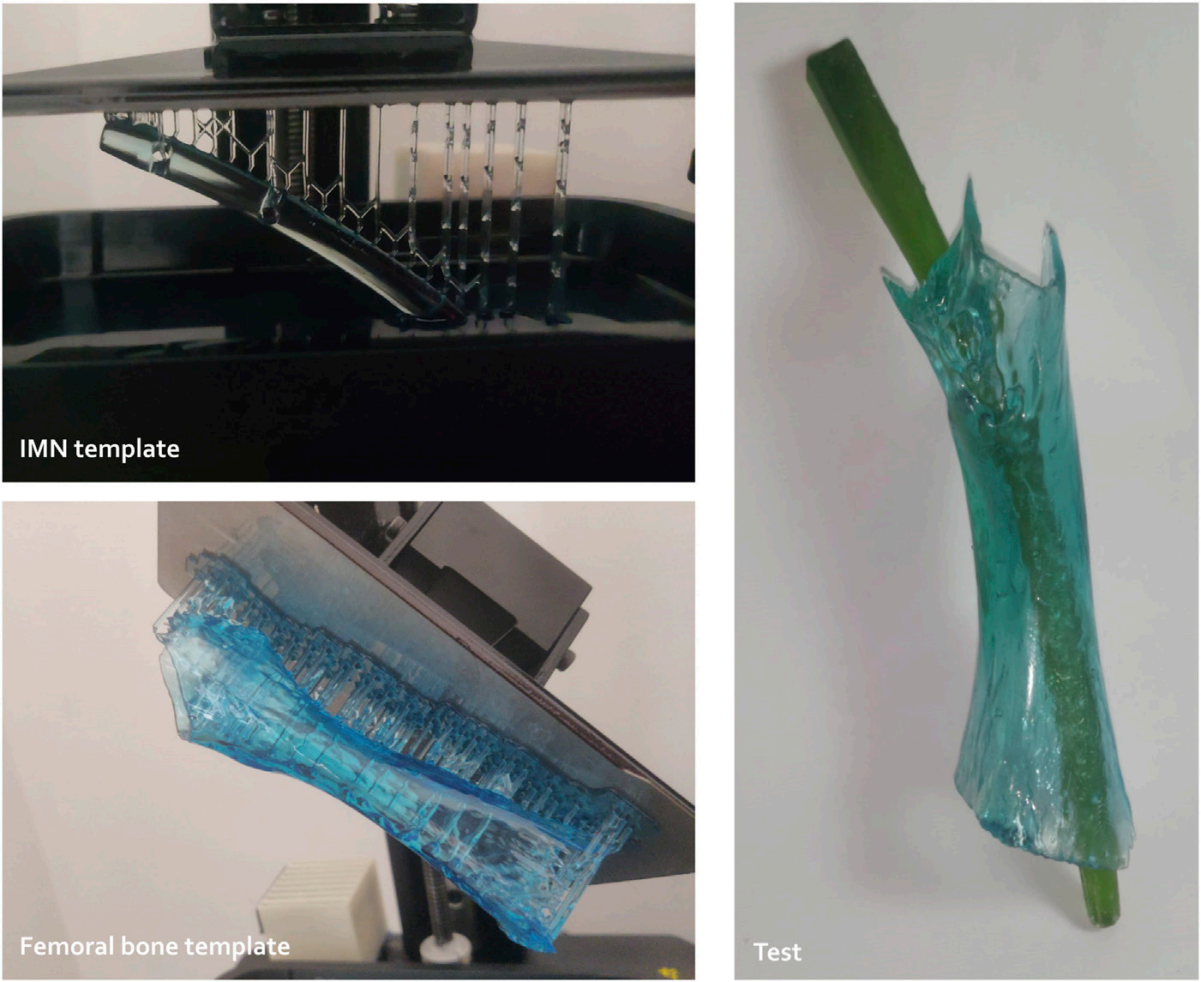
Preliminary tests to study the conceptualization of custom-made intramedullary nailing (IMN) including resin template printing and trials (3D Materials SuperPP resin and Anycubic resin for the nail and femoral templates, respectively).

The femoral IMN was custom-designed using Autodesk Fusion360 CAD software. 3D-printed templates were successively improved by *in vitro* testing on synthetic bones (Figure 1). Those bones were 3D-printed in resin (Standard Clear resin, Anycubic^®^) using MSLA (masked Stereolithography), based on CT scans and allowed to adjust and achieve the final nail design.

#### 2.3.1 Overall design

The general design of the centromedullary nail consists of a curved cylinder with a tapered and rounded distal end to facilitate insertion. The curvature and diameter of the nail are determined based on anatomical studies. A solid design was chosen, which does not allow for the passage of a guide wire in the center of the nail. While this prevents the use of a guide wire, it enables the nail to be more resistant (Gkouvas et al., 2019). Effectively, a smaller diameter than those typically used in humans, associated with a central tunnel for the guide would increase the risk of weakening the final device. The position, number, and type of locking screws are also determined based on anatomical studies and the literature discussing the benefit of a higher number of static locking screws (Horn et al., 2009; Freeman et al., 2011; Li et al., 2020; Martínez-Aznar et al., 2023).

#### 2.3.2 Associated tools

**FIGURE 2:**
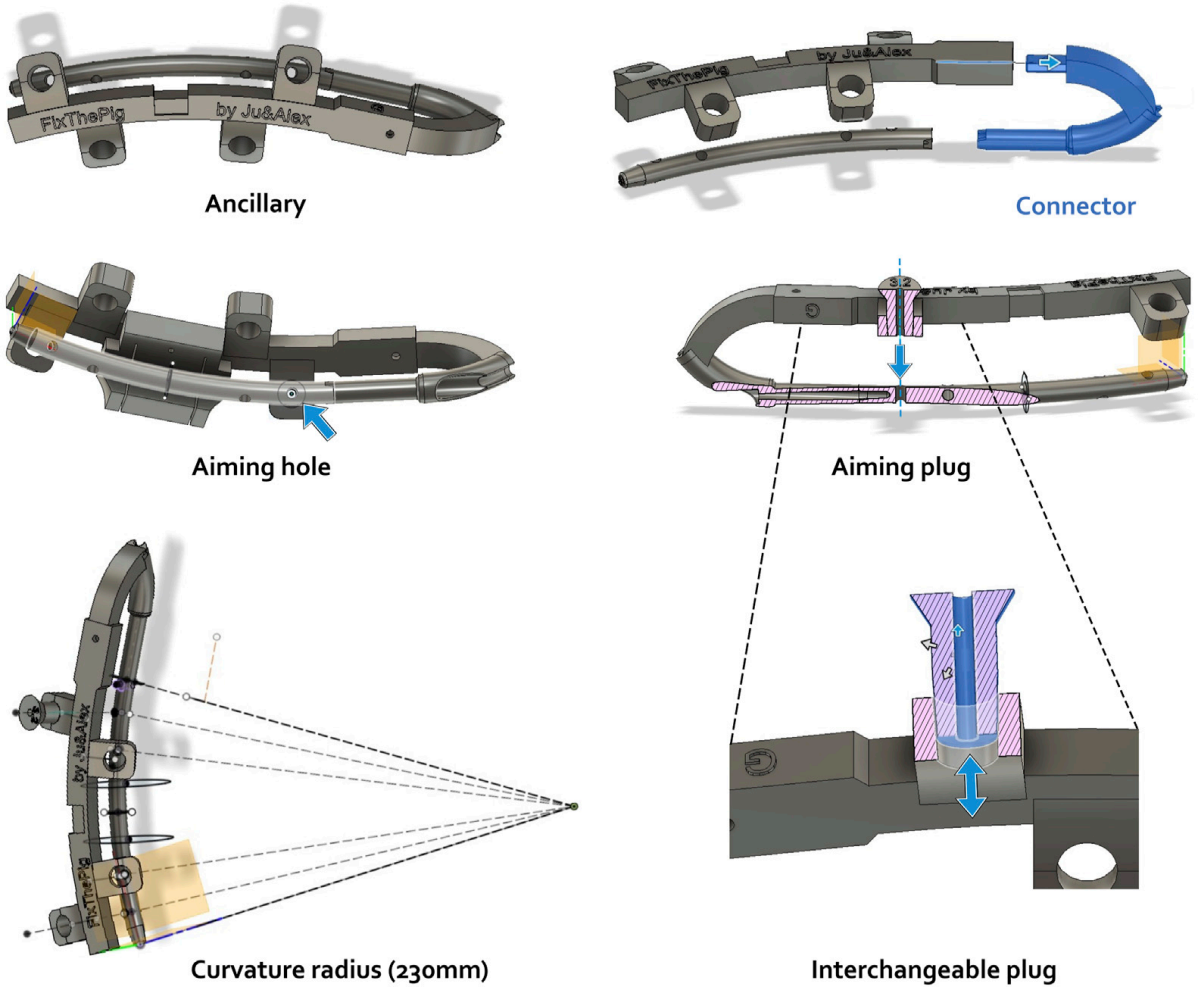
Ancillary modulization using Autodesk Fusion360 CAD software including distinct tools (ancillary, connector, plug) and their aiming function. Actions of aiming hole, aiming plug and interchangeable plug are illustrated by blue arrows. Pink hatched area: frontal section.

A variety of instrumentation, including guide instruments, have been developed for use with the IMN. Figure 2 shows the various elements presented in this section.

The first component, designed to be printed in metal and attached to the nail, consists of a connector that screws onto the nail. This connector has a hook-like shape that allows for a good grip of the nail during insertion into the femur and provides an anchorage point for the accessory. To facilitate attachment to the nail, a blind hole has been incorporated proximally on the nail. The diameter of this hole is designed to accommodate an M4 screw after tapping. A locking system was also implemented to prevent rotational movement between the connector and the nail once they are screwed together. The portion of the connector that attaches to the nail has the same diameter as the nail, allowing it to enter the bone through the same opening, thereby allowing the nail to be inserted without protruding. The other end of the connector is designed to fit into the ancillary device described below. Fixation is achieved with an M3 screw that is placed perpendicular to and through the connector.

The ancillary device is designed to be 3D-printed in polyamide, resulting in a thicker structure. It has a series of appendages aligned with the axes of the locking screws of the centromedullary nail. These appendages feature slightly tapered holes, to accommodate interchangeable plugs that match the diameters of the various surgical drill bits required during the procedure.

The plugs are also designed to be 3D-printed in polyamide and have a conical shape that complements the holes in the ancillary device’s appendages. This design enables the plugs to fit securely while maintaining their axis.

To facilitate a standardized creation of CSBDs for research purposes, a symmetric double osteotomy can be achieved using a custom-designed cutting guide component. This component is engineered to clip into a notch on the ancillary device, ensuring precise and symmetric osteotomies between the screw fixations, thereby enabling reproducible CSBDs.

To enable bone cutting once the guide is positioned relative to the nail via the ancillary, the nail must be temporarily removed. To achieve this, holes in this cutting guide are strategically positioned to allow the placement of K-wires onto the bone, securing the cutting guide to the bone and permitting the temporary removal of the ancillary and nail to perform the osteotomy.

### 2.4 Production and post-processing

The IMN was 3D-printed in titanium ( ${Ti}_{6}{Al}_{4}$ V) by Materialise NV (Leuven, Belgium) using Direct Metal Laser Sintering (DMLS) technology. The connector was fabricated in stainless steel via DMLS 3D-printing. All aiming tool components were produced in polyamide 12 (PA 12) using Selective Laser Sintering technology from Materialise NV (Leuven, Belgium). The PA 12 material used in the experiments was compliant with the ISO 10993-5 standard for cytotoxicity (Materialise, 2024). After the printing, post-processing steps included tapping the proximal end of the nail to fix the connecting screw between the nail and the connector, and removing the excess material for the plug holes and screw holes in the nail in order to achieve a good fitting of the various elements, without excessive friction.

### 2.5 Experimental study

Following preliminary *in vitro* assessments of the nail’s size and shape, as described in Section 2.3, we proceeded to *in vivo* testing in a large animal model to evaluate the bone healing process, as, to the best of our knowledge, there is no *in vitro* test that allows for accurate modelling of the bone healing process which necessitates biological *in vivo* response.

#### 2.5.1 Porcine model

Female Aachen minipigs ( N=4 , 40−55 kg, Carfil Quality, Oud-Turnhout, Belgium) were used as a large animal model for CSBD creation and fixation (Pawlowsky et al., 2017). This study was approved by the local ethics committee (Ref 2023/UCL/MD/39, approved on the 18th of July 2023). Each minipig was housed in our facilities for a minimum of 2 weeks before the surgery for acclimatization. Each animal was operated on both femurs.

#### 2.5.2 Surgical procedure

The minipig was installed in ventral decubitus on a specific table to allow bilateral one-step surgery, under general anaesthesia (induction with an IM injection of 6 mg/kg Zoletil 100^®^ (Virbac, Carros, France) and 2 mg/kg Rompun^®^ (Bayer, Leverkusen, Deutschland), followed by endotracheal ventilation with Isoflurane ( 0−1.5% , Forene^®^, AbbVie, Wavre, Belgium)). An intrajugular central line was set up to administer curare, appropriate analgesia, and anti-inflammatory drugs, and provide post-operative analgesia. A 2 g bolus of Kefzol^®^ (Natrium Cefazoline, Sandoz^®^ Sol Inj, Novartis Company) was also administered at induction, followed by 1 g Kefzol every 3 h during surgery. The hindquarter was washed with isobetadine soap, gently shaved, and disinfected with hydroalcoholic isobetadine. Classical surgical drapes allowed a sterile procedure. The surgical approach used a pure lateral incision centred on the femoral diaphysis, proximally extended for the proximal entry point of the nail in the greater trochanter. The intramedullary canal was reamed until a diameter of 9 mm. The nail, connected with the ancillary, was inserted and its position was verified with fluoroscopy. The fixed cutting guide allowed the creation of a CSBD of 2.5 cm long in the mid-shaft of each femur, centred to the nail. To ensure the reproducibility of the defect, all massive bone allografts (MBA) were scanned to detect morphological discrepancies between the donor and host femurs. They were transplanted successively to the following minipig respecting the laterality. Then, the nail was inserted again through the MBA in between both autologous femoral extremities and locked by fixation screws thanks to the ancillary and aiming plugs.

#### 2.5.3 Post-operative follow-up

A post-operative fluoroscopy was performed immediately after the surgery to validate the osteosynthesis. Every 2 weeks, each minipig underwent standard X-rays (DigitalDiagnost, Philips, France) and a CT scan (beam collimation 0.6 mm, tube voltage 140 kV) until 3 months of follow-up. These imaging techniques allowed to verify potential material protrusion, displacement or breakage, consolidation, and healing kinetics.

#### 2.5.4 Explant analysis

One minipig was euthanized just after the surgery to harvest MBA, another after 6 weeks, and the last two minipigs were euthanized after 3 months (IV bolus injection of T61, 0.2 mg/kg). Both femurs of the last three minipigs were fully explanted with the IMN in place to assess the callus formation and to give a manual mechanical appraisal of consolidation.

## 3 Results

### 3.1 Design of the 3D-printed IMN

#### 3.1.1 IMN featuring depending on anatomical study

**FIGURE 3:**
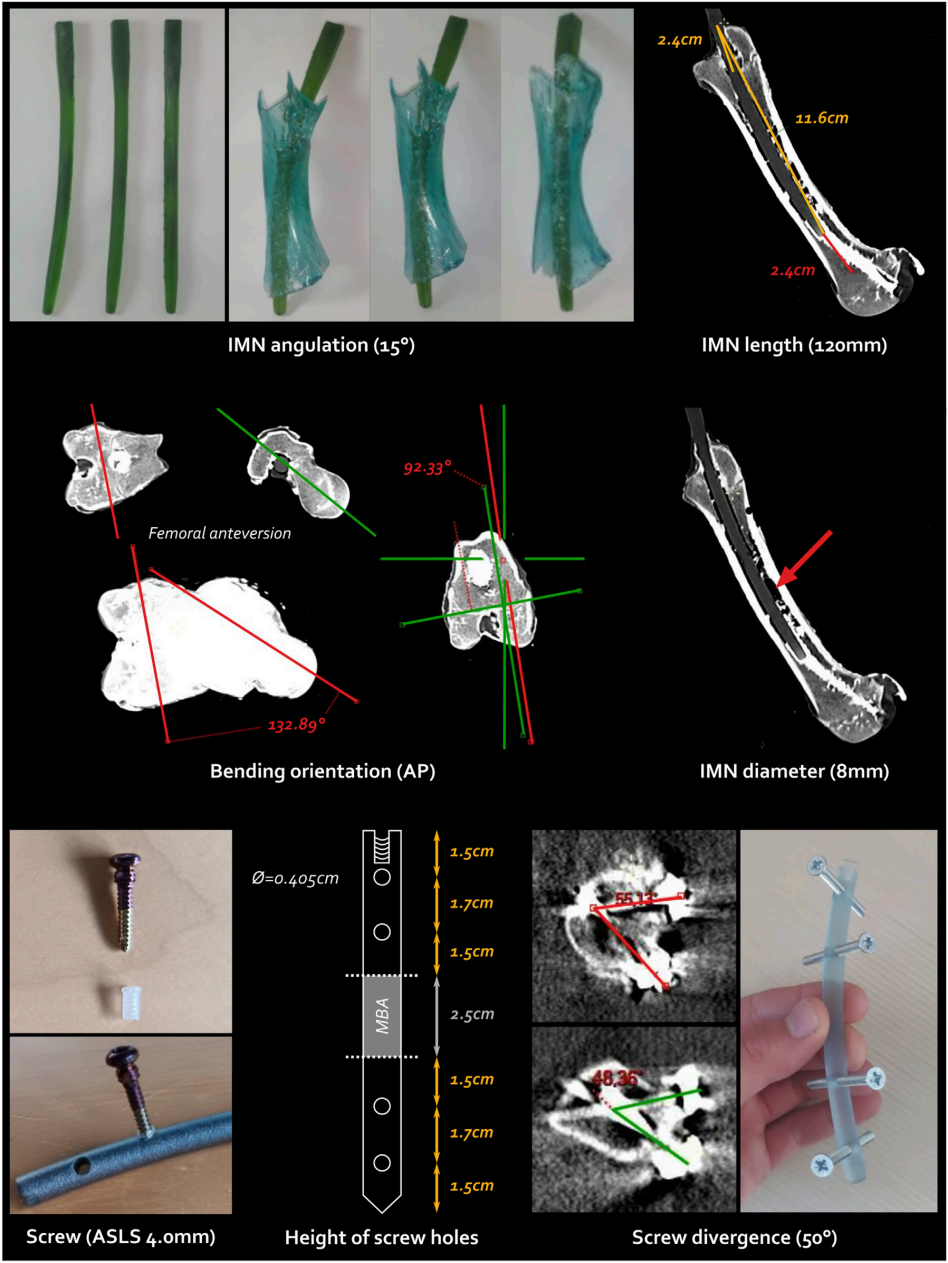
Every single step and feature of the intramedullary nailing (IMN) conception. The screw type is the ASLS screws illustrated at the bottom left, the cross-headed screws shown at the bottom right are pre-test screws. AP, anteroposterior; ASLS, angular stable locking system; Ø, diameter.

Every IMN feature, derived from the reported femoral anatomy measurements, is illustrated in Figure 3.

##### 3.1.1.1 IMN bending and orientation

First, a femoral bending of 15° was calculated on CT scans. The IMN then featured a radius of curvature of 230 mm. The insertion of the IMN test into an *in vitro* femur, through the intended entry point at the tip of the greater trochanter, led us to ascertain the spontaneous orientation of the IMN. Naturally, the IMN positioned itself with its curvature along the sagittal axis, with the concave curve oriented towards the posterior aspect. This alignment was corroborated by the CT scans, which matched the femoral anteversion (±130°) . This orientation further helped to create the ancillary in an orthogonal plane (90°) compared to the bending axis.

##### 3.1.1.2 IMN length and diameter

Including this bending, the total IMN length and diameter were determined as 120 and 8 mm, respectively to fit at best the intramedullary canal.

##### 3.1.1.3 Screw type

Angular Stable Locking System (ASLS, ⓒ Depuy Synthes, Johnson & Johnson) screws were selected to ensure both angular and axial stability within the metaphysis due to the hourglass-shaped diaphysis while reducing the total number of screws (Horn et al., 2009; Wähnert et al., 2012; Wähnert et al., 2013; Lenz et al., 2013). This system incorporates three distinct core diameters in the screw, along with a resorbable plastic sleeve that expands into the nail with the second diameter of the screw, enhancing angular stability.

##### 3.1.1.4 Number and height of screw holes

Another benefit of the bending orientation lies in the placement of lateral locking screws, in agreement with the direct lateral surgical approach. The increased stability offered by ASLS screws allowed for the use of only two bicortical screws on each side of the CSBD. Four holes strategically placed along the nail allowed for screw fixation via a lateral approach. Careful attention was paid to the symmetric positioning of the hole heights to prevent any weak points, ensuring the ostectomy was precisely centred within the fixation.

##### 3.1.1.5 Screw divergence

A maximal divergence angle of 50° was applied, considering the space available within the surgical approach, to further enhance mechanical stability.

#### 3.1.2 Final design of 3D-printed IMN, aiming tools and cutting guide

**FIGURE 4:**
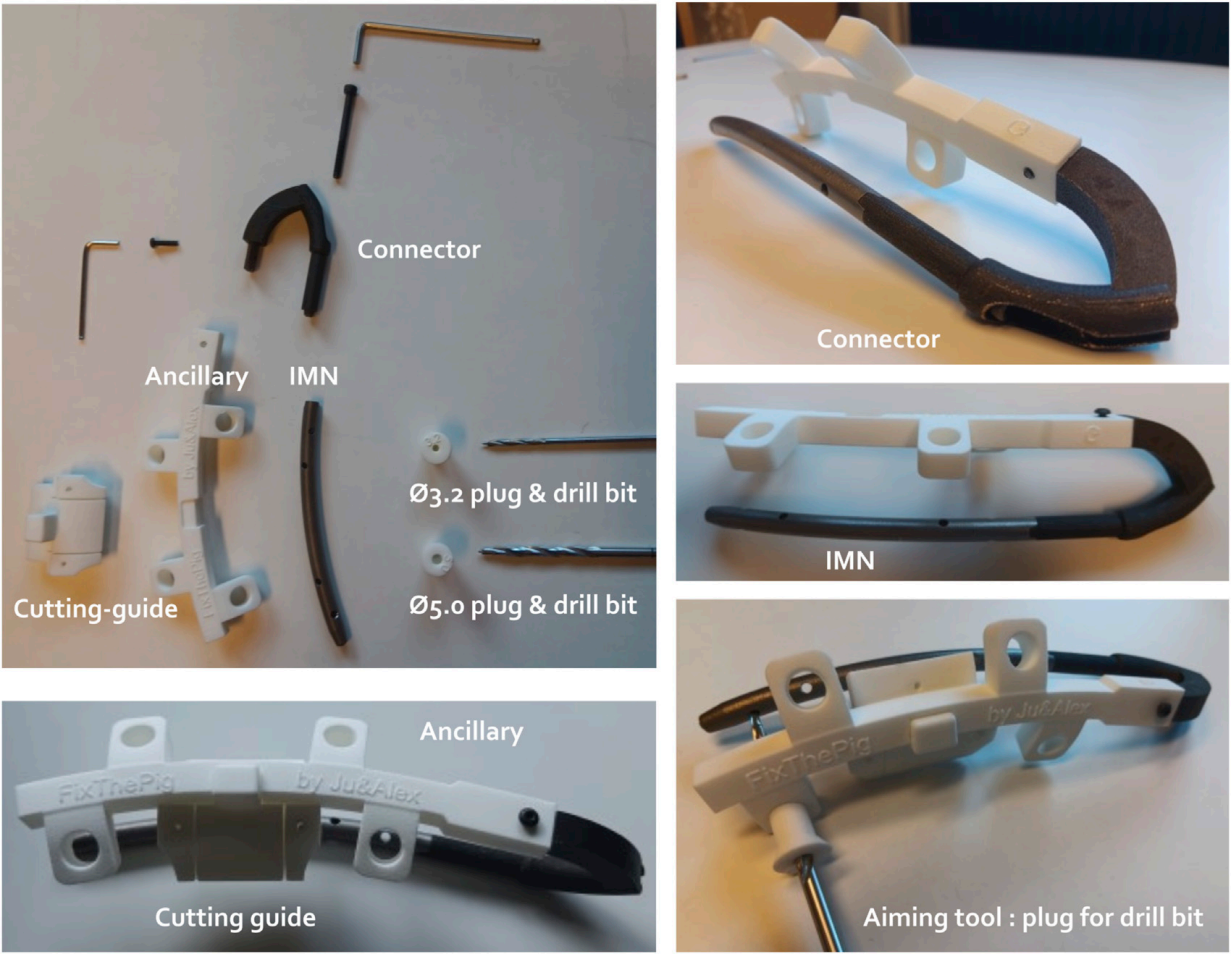
Different components of the intramedullary nailing (IMN) osteosynthesis. The IMN is connected with the ancillary through the connector. The ancillary unit holds the plugs of different diameters (Ø) to guide respective drill bits to the hole of the IMN. A cutting guide is designed to ensure the centralization of both osteotomies and can be clipped on the ancillary.

All the 3D-printed components including the IMN, the stainless steel connector, the ancillary, various plugs, as well as the cutting guide fixed on the ancillary are shown in Figure 4.

### 3.2 *In vivo* implantation

**FIGURE 5:**
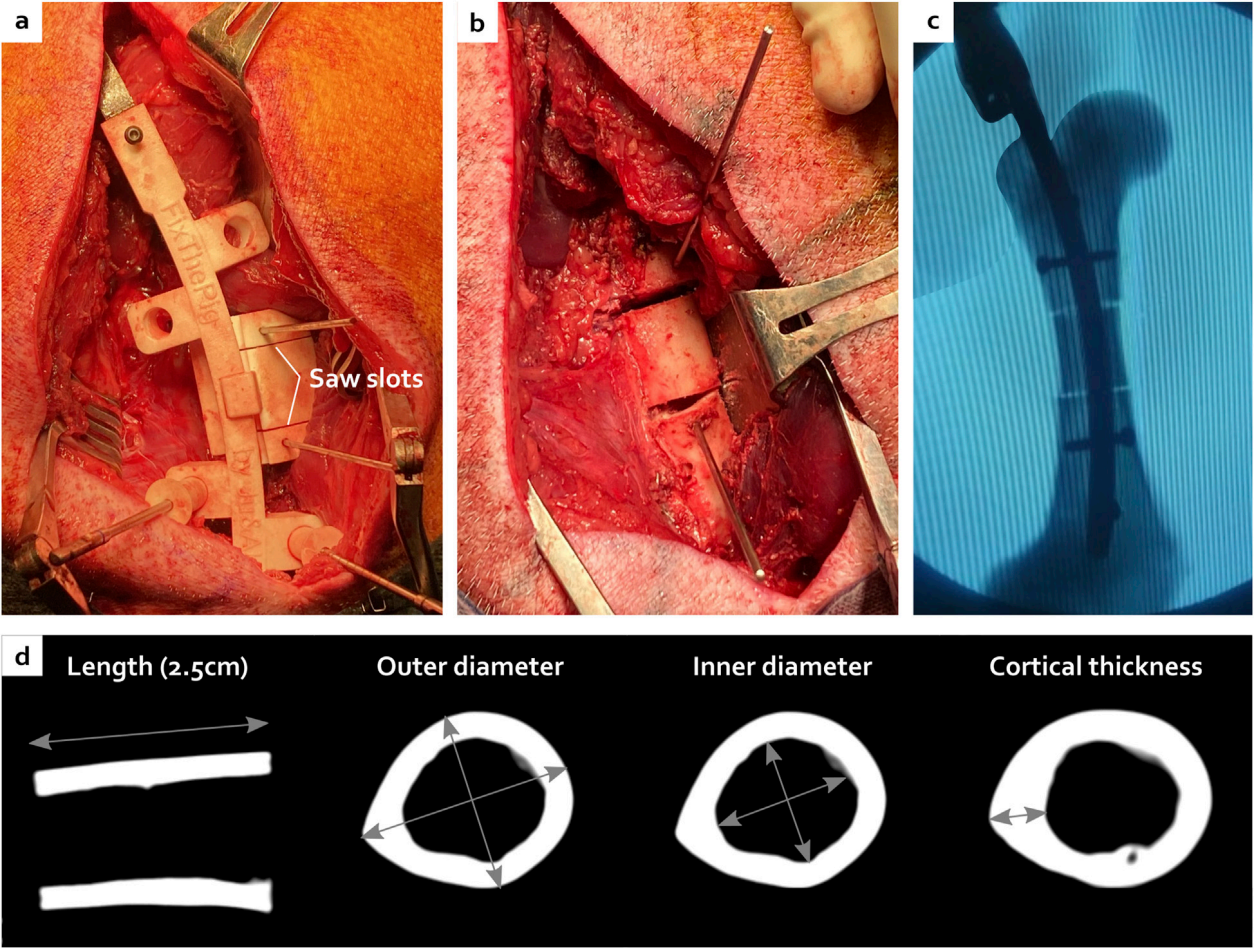
Creation of the critical-size bone defect with the ancillary and the cutting guide **(A)** leading to a centred ostectomy with reproducible morphology visible during the surgery **(B)**, under fluoroscopy **(C)** and after CT scan analyses **(D)**.

**FIGURE 6:**
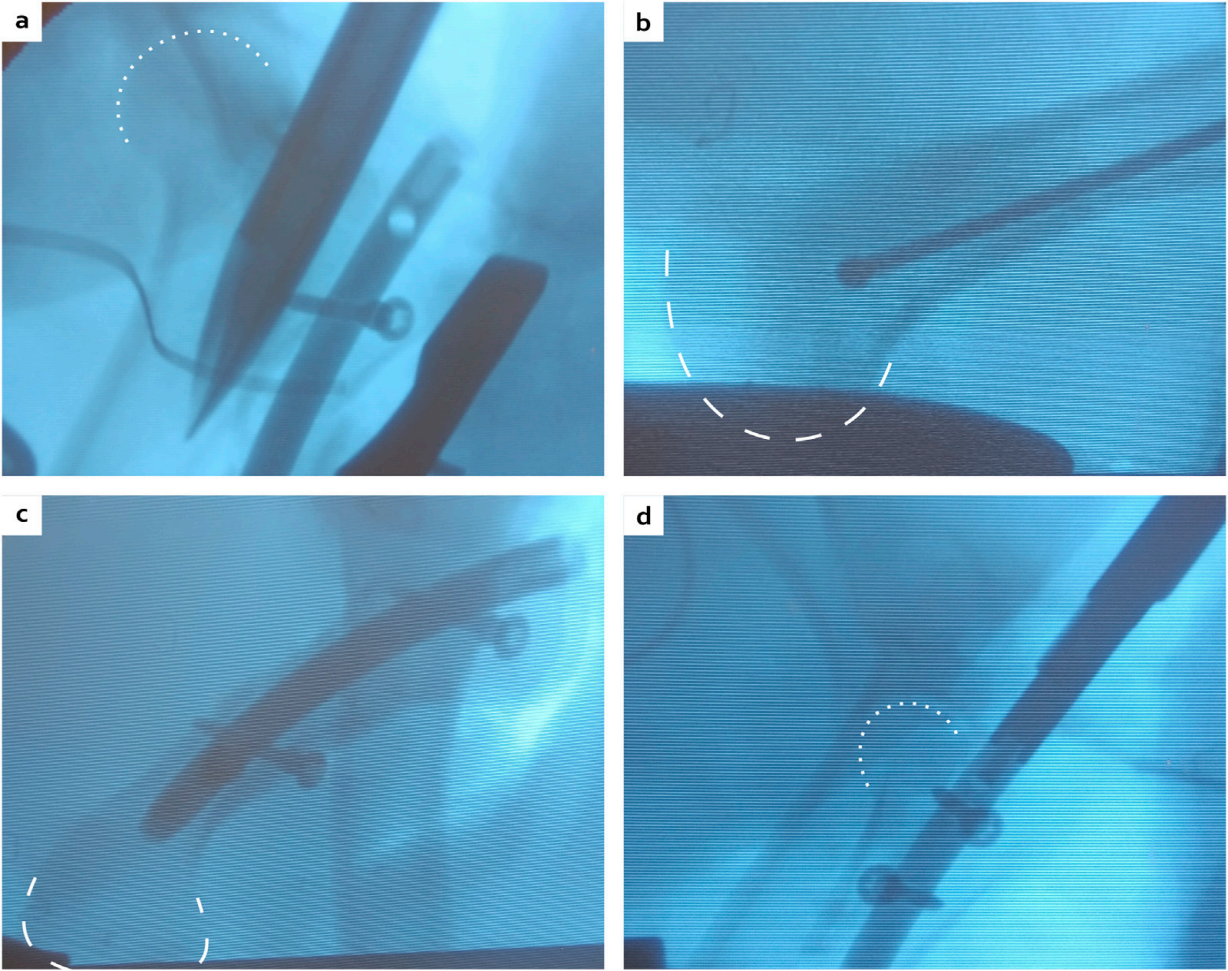
Peroperative fluoroscopies showing the physis passage with a straight square awl **(A)**, the maximal distance reached by the ball-tipped guide wire **(B)**, the final construct with the nail, two distal locking screws and only one proximal locking screw **(C)**, or the double proximal locking screws **(D)**. White dotted lines: femoral head contour, white dashed lines: condyle contour.

During the surgeries, the ancillary and the cutting guide successfully allowed a symmetric double osteotomy. The ostectomy was easily performed and allografts were very reproducible without major morphology discrepancy based on subsequent CT scans analyses (Figure 5), similar to anatomical measurements described in Section 2.1. The proximal and distal metaphyses were very hard areas to go through. The proximal metaphysis was successfully permeabilized using a straight square awl to a depth of 5 cm (Figure 6A). However, no surgical instrument was able to penetrate the distal epiphysis (Figure 6B). Therefore, of the three minipigs included in the follow-up, two received only one proximal screw as the nail was not fully inserted (Figure 6C). The third one received two proximal screws as planned (Figure 6D).

### 3.3 Bone healing and consolidation with the custom-made IMN

**FIGURE 7:**
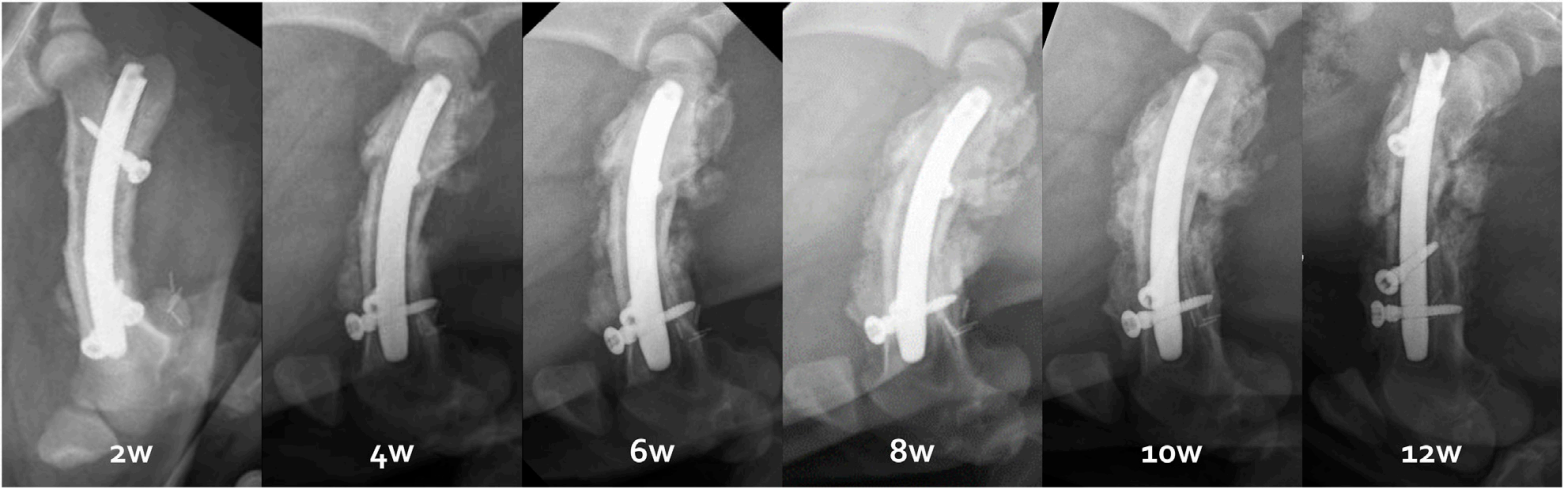
Radiological follow-up from 2 to 12 weeks illustrated by the right femur evolution of the third minipig.

**FIGURE 8:**
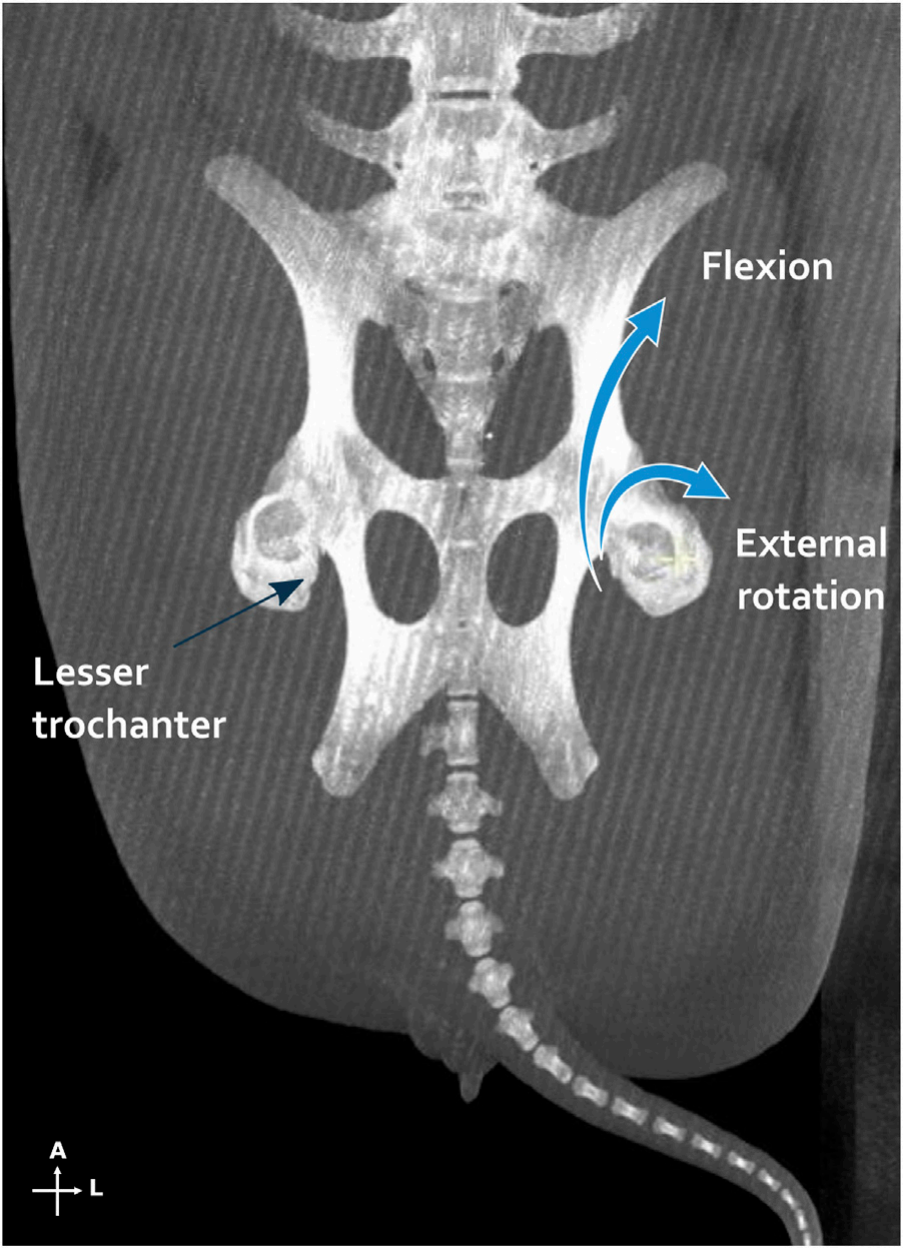
Iliopsoas muscle action illustrated by a maximum intensity projections on the multiplanar reconstruction of both femurs and pelvis. On the left side, the tendon insertion point is shown on the lesser trochanter, while on the right side, the action of the muscle is schematized. A, anterior; L, lateral.

X-rays and CT scans showed that the distal osteotomy was always perfectly reduced and fixed (Figure 7). However, for all 6 femurs, the proximal fragment was displaced in a flexed position and external rotation due to the action of the iliopsoas muscle (Figure 8). This proximal fixation with only one screw led to hypertrophic callus suggesting pseudarthrosis in 3 of the 6 femurs. The one minipig receiving two proximal screws had to be euthanized at 6 weeks because of both sides proximal femur fracture.

### 3.4 Explantation

**FIGURE 9:**
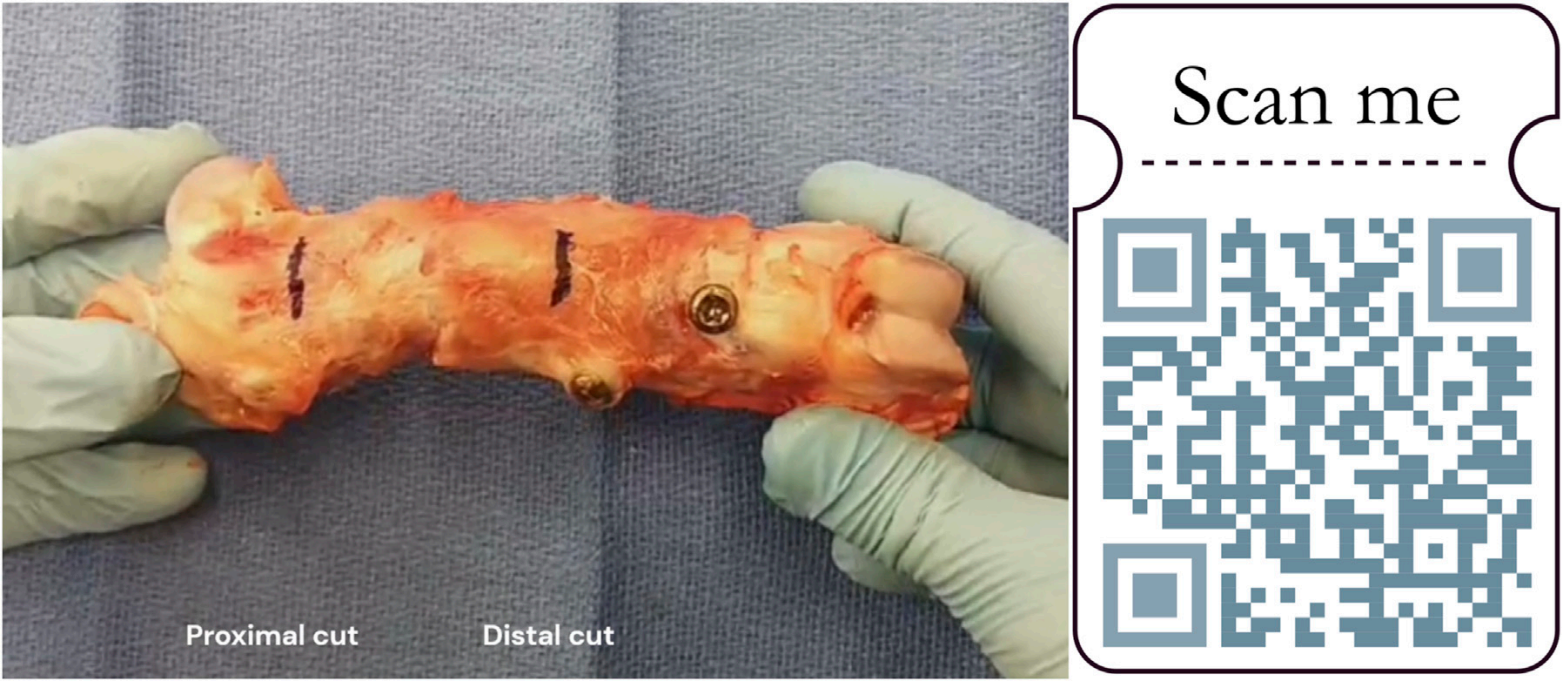
Manual stability testing of a femur explant. Black marks located the further cut (1 cm proximally and distally from both osteotomy junctions). https://zenodo.org/records/11061177.

Manual examination of femoral explants first confirmed pseudarthrosis on half of the proximal osteotomy junctions. Concerning the distal junction, only one right femur was manually not stable after 12 weeks. Every other distal junction was manually stable after 6 or 12 weeks (Figure 9).

## 4 Discussion

This study proposed a new concept of 3D-printed IMN for preclinical large animal models such as minipigs. The utilization of ancillary tools and cutting guides facilitated the achievement of a symmetric double osteotomy during surgeries. Moreover, the ostectomy process was executed smoothly, resulting in allografts with highly reproducible morphology. These findings underscore the efficacy of the surgical approach and the reliability of the instruments used.

However, the 3D design could still be slightly improved. Notably, during the surgical procedure, the ancillary was found to be susceptible to deformation by surrounding muscles. This can be linked to the deliberate placement of the ancillary in close proximity to the bone, which was intended to enhance stability of the overall assembly and improve the precision of the osteotomy. This placement should be revised to increase the distance between the ancillary and the bone, in addition to making the ancillary more rigid.

A comparative analysis with commercial intramedullary nails, such as the Stryker^®^ T2 or Synthes^®^ FRNA) could provide valuable insights for future design improvements. Notably, these devices often feature an ancillary component that is offset or more eccentric relative to the bone, allowing for a less invasive surgical approach that can be performed outside of the muscle tissue. However, in the specific context of preclinical bone defect creation, this design aspect must be balanced with the benefits of having a cutting guide positioned closer to the bone, which can provide more precise control. Concerning the implants, manual examination of femoral explants confirmed the occurrence of pseudarthrosis in some instances at proximal osteotomy junctions. Nevertheless, all distal junctions appeared consolidated upon manual testing, except one. Certain outcomes may hinge on technical refinements, particularly concerning enhancing mechanical stability. Macroscopic evaluations via X-rays and CT scans revealed perfect reduction and fixation at distal osteotomy sites, juxtaposed with challenges encountered in maintaining consistent reduction at proximal fragments. This imperfect alignment often resulted in hypertrophic callus formation, as evidenced by cases receiving only one proximal screw. In a severe case, proximal femoral fractures necessitated the euthanasia of the pig receiving two proximal screws. As with human femoral nails, the entry point is crucial for the successful placement of the nail (Charopoulos and Giannoudis, 2009; Robles et al., 2022; Alzahrani et al., 2023). In this study, human anatomical landmarks were adapted for use in the porcine femur through radiological anatomical studies. The nail must be initially inserted before creating the osteotomy, allowing for optimal positioning relative to the intact femoral anatomy. This approach minimizes the risk of poor reduction that can result from an improperly located initial entry point, as often seen in femoral fracture reductions. Mechanically, a single proximal screw may permit excessive mobility at the osteotomy site, while two screws might render the construct overly rigid and misaligned with forces transmitted during weightbearing, thereby increasing the risk of fracture. Similar to commercial intramedullary nails, incorporating a slotted hole distally could enable nail dynamization, facilitating improved compression and potentially enhancing the healing process. Potential adaptations could involve employing a reduction clamp to stabilize the reduction prior to fixation, shortening the IMN length to accommodate two screws below or at the level of the femoral neck, or designing the upper screw as a cephalic screw to better distribute weightbearing forces, akin to practices in human with proximal femoral nails used for pertrochanteric fractures. Furthermore, in this study, the loss of bone substance created by the saw blade is not replaced as a graft is transplanted from one pig to another successively. This millimeter loss of height could slightly shorten the femoral length and accentuate the proximal protrusion of the nail. The addition of a mechanical compression system on the nail or the ancillary could prove advantageous in enhancing interfragmentary contact and compression, especially considering the challenges of manual compression during these procedures. Using a sigmoid or step cut technique on at least one side of the MBA could help minimize rotational instability and increase the surface area of bone contact (Cascio et al., 2003; Gundavda et al., 2023).

Furthermore, although potentially less rigid (Gkouvas et al., 2019), titanium alloy hardware in IMN procedures minimized artifacts, thereby enhancing the accuracy of imaging analyses compared to plates or external fixation methods, or other alloys (Buckwalter et al., 2011).

In investigations concerning substantial bone defects, the pig model has proven notably advantageous when compared to sheep and goats or rabbits (Klein et al., 2020; Viateau et al., 2006; Pearce et al., 2007; Reichert et al., 2009), particularly in terms of bone mineral density, anatomy, structure, morphology, healing process, and remodelling (Reichert et al., 2009). Even with its plexiform predominance over dense Haversian structure (Reichert et al., 2009), porcine bone shows the closest resemblance to human samples (Aerssens et al., 1998; Pearce et al., 2007; Reichert et al., 2009). The main issues with the pig model are its ease of handling and its high growth rates (Reichert et al., 2009; Pearce et al., 2007), why minipig was proposed. Since the IMN production was based on a 3D-printed titanium scaffold, the design could be easily adapted to all different bones or animal species according to a preliminary anatomical analysis.

Additionally, the IMN design allows for precise and reproducible CSBD creation and treatment keeping surrounding soft tissues intact. Studies focusing on osteogenic membrane wrapping a CSBD could benefit from an accurate placement of the membranes, ensuring uniform coverage and interaction with the filling bone graft, without being compressed by plates. By facilitating a controlled environment, the IMN system enables a more accurate assessment of the membrane efficacy in enhancing bone regeneration and provides a reliable tool for future investigations into optimizing guided bone regeneration techniques.

In conclusion, this study demonstrated the feasibility of 3D designing an IMN osteosynthesis for preclinical large animal models, like Aachen minipigs, and the ability to multiply surgical techniques and tools according to the specific needs of rigorous and demanding research, such as the implantation of an MBA and a surrounding osteogenic membrane.

## Data Availability

The raw data supporting the conclusions of this article will be made available by the authors, without undue reservation.

## Abbreviations

CSBDs, critical size bone defects; IMN, intramedullary nailing; CT, computed tomography; MBA, massive bone allograft; ASLS, angular stable locking system; CAD, computer-aided design.
